# Ionic Cross-linked Chitosan Beads for Extended Release of Ciprofloxacin: *In vitro* Characterization

**DOI:** 10.4103/0250-474X.40326

**Published:** 2008

**Authors:** A. Srinatha, J. K. Pandit, S. Singh

**Affiliations:** Department of Pharmaceutics, Institute of Technology, Banaras Hindu University, Varanasi - 221 005, India

**Keywords:** Beads, chitosan, ciprofloxacin, ionic cross-linking, tripolyphosphate

## Abstract

Chitosan beads loaded with ciprofloxacin hydrochloride were fabricated by ionic cross-linking with sodium tripolyphosphate. The beads showed an excellent water retention property. The degradation of fabricated beads was influenced by the pH of test medium. High drug load was achieved within the bead with a short curing time. Drug release was high in acidic medium (pH 1.2) vis-à-vis intestinal medium (pH 7.4). Ciprofloxacin hydrochloride release increased with an increasing concentration of ciprofloxacin and decreasing proportion of chitosan. Drug release followed both first-order and Higuchi's root time kinetics showing non-Fickian release mechanism.

An ideal drug delivery system should release the drug in the right body compartment at a rate required for a specific treatment. Multiple unit solid dosage forms distribute the drug load more uniformly in the gastrointestinal tract with the aim to reduce local irritation^1^,^2^. Chitosan, a naturally occurring polysaccharides, has received major attention in drug delivery systems. It is biocompatible and non-toxic. Chitosan is a weak cationic polysaccharide composed of [a (1→4) 2-amino-2-deoxy-β-D-glucan] obtained by the alkaline deacetylation of chitin. Chitosan spheres are usually prepared by precipitation^3^, anionic cross-linking^4^, chemical cross-linking^5^, thermal cross-linking^6^, co-acervation^7^ and emulsification ionic-gelation^8^ methods.

Ciprofloxacin, a fluoroquinolone was used as the model drug for the study. It has high bactericidal activity against uropathogens and in the treatment of urinary tract infections with a biological half-life of 4 h. It is advised in complicated intra-abdominal infections (in combination with metronidazole), infectious diarrhoea, typhoid fever (enteric fever), and uncomplicated cervical and urethral gonorrhoea. Ciprofloxacin is beneficial in treatment of mild to moderate Crohn's disease and in maintenance of remission^9^, and is the first line of treatment in treating gastrointestinal anthrax caused by *B. anthracis*^10^ where the death range is 25-60%^11^. Extended-release formulation of ciprofloxacin provides systemic drug exposure comparable with that achieved with twice-daily administration of conventional, immediate-release ciprofloxacin, while also attaining higher maximum plasma concentrations with less inter-patient variability^12^.

The aim of the present investigation was to evaluate the factors influencing the physical properties of the chitosan beads. The study involved characterization of water uptake of the beads, its behaviour in acidic and alkaline media, and influence of formulation and study variables on the drug release.

## MATERIALS AND METHODS

Ciprofloxacin hydrochloride was a generous gift of M/s Dr. Reddy's Laboratories (Hyderabad, India). Chitosan (Mol. wt. 2 × 10^5^, degree of deacetylation 82-88%) was obtained from M/s Kraeber Gmbh and Co (Ellerbek, Germany). Sodium tripolyphosphate (TPP) was procured from S. D. Fine Chemicals (Mumbai, India). All other reagents were of analytical grade and used as received.

### Preparation of chitosan beads:

Chitosan beads were prepared by the ionic cross-linking as described by Bodemier *et al.*^7^ with minor modifications. Briefly, chitosan was dissolved in acetic acid (2% v/v) and stirred for 6-7 h on a magnetic stirrer (Remi Equipments, Mumbai). Weighed amount of ciprofloxacin HCl (Table 1) was added to the polymeric solution and stirred on a magnetic stirrer for 2 h, and allowed to stand till the removal of the entrapped air bubbles. The pH of the drug-chitosan solution was adjusted to pH 4-4.5 with dilute alkali solution (0.1 M, NaOH). Chitosan solution containing drug was added dropwise using a syringe fitted with a flat-end needle (23G, 0.7 mm id) into sodium tripolyphosphate solution (1-4% w/v, pH 5, 60°). Beads were left for 20-30 min, unless otherwise specified, and after curing, were collected by filtration, washed twice with distilled water and dried at 50° for 4 h and then at room temperature (25°) for 12 h.

**TABLE 1:** FORMULATION COMPOSITIONS, PHYSICAL AND RELEASE CHARACTERISTICS OF THE FABRICATED BEADS

Batch	Chitosan (% w/v)	Ciprofloxacin (% w/v)	TPP (% w/v)	Wet bead size (mm)	Dry bead size (mm)	IE (%)	Release exponent (n)
CB1	2	1	1	2.21 ± 0.32	0.61 ± 0.05	67.30 ± 2.34	0.51
CB2	2	1	2	2.23 ± 0.19	0.60 ± 0.09	67.21 ± 1.95	0.53
CB3	2	1	3	2.22 ± 0.09	0.62 ± 0.14	69.08 ± 3.11	0.53
CB4	2	1	4	2.23 ± 0.17	0.61 ± 0.08	68.70 ± 1.61	0.58
CB5	1	1	2	2.08 ± 0.51	0.65 ± 0.15	60.53 ± 2.09	0.51
CB6	3	1	2	2.31 ± 0.24	0.70 ± 0.03	73.67 ± 2.44	0.58
CB7	2	0.5	2	2.11 ± 0.21	0.69 ± 0.11	75.80 ± 2.39	0.59
CB8	2	2	2	2.24 ± 0.28	0.68 ± 0.08	64.42 ± 3.10	0.47

IE- Incorporation efficiency, calculated from the percentage ratio of actual drug concentration in the bead to theoretical drug concentration

### Particle size determination:

The particle size of the prepared beads in a sample was measured with an optical micrometer fitted with a calibrated eye piece. The mean of 100 beads was noted as particle size. The sizes of both wet and dried beads were measured. All readings are average of three trials ± SD.

### Determination of encapsulation efficiency:

About 50 mg of the beads were crushed in a glass mortar and digested in 0.1 N hydrochloric acid (pH 1.2) for 24 h in a graduated flask. The solution was filtered through a G-2 filter and an aliquot was used to assay for drug content spectrophotometrically (Jasco 7800, Japan) at 276 nm against a suitable blank. The encapsulation efficiency was calculated by expressing the actual entrapment level divided by the theoretical entrapment level, as a percentage. The values are average of three trials ± SD.

### Surface morphology of the beads:

The surface morphology images were obtained by scanning bead surface on an electron microscope (SEM, Philips XL20, Holland) under vacuum. Beads were mounted on brass stubs using silver paste and scanned under vacuum at the required magnification at room temperature.

### Water uptake studies:

Water uptake capacity of the beads was determined in 0.1 N HCl (pH 1.2) and pH 7.4. A weighed quantity of beads (100 mg) was immersed in SGF (0.1 N HCl, pH 1.2) and SIF (pH 7.4), and at regular intervals of time, the beads were reweighed after carefully wiping off excess of liquid with a tissue paper. The water uptake was determined from the expression; (W_t_ – W_o_)/W_o_, where, W_t_ and W_o_ are the weight of the beads at time‘t’ and under dry state, respectively.

### *In vitro* degradation study:

*In vitro* degradation of beads was investigated in pH 1.2 and 7.4. Beads (100 mg) were placed in basket of USP-XXIII dissolution apparatus containing 900 ml of respective media at 50 rpm. At regular intervals beads were removed, excess liquid was wiped off, and oven dried at 80° till constant weight. The mass loss of the beads was calculated from the dry weights measured at the beginning and predetermined time intervals.

### *In vitro* drug release studies:

*In vitro* release of ciprofloxacin from the beads was performed in USP XXIII dissolution apparatus II with a paddle speed of 50 rpm. The dissolution medium was 900 ml of SGF without enzyme (0.1 N HCl, pH 1.2) for first 2 h and subsequently rest of the release study was performed in SIF (phosphate buffer, pH 7.4) at 37 ± 0.2°. At regular time intervals, 5 ml aliquot was withdrawn and replenished with an equal volume of fresh dissolution medium. The drug content in the aliquot was assayed spectrophotometrically at 276 nm (Jasco 7800, Japan). A study was performed concurrently with placebo beads to record for any interference by the bead components.

For analyzing drug release kinetics, *in vitro* release data were fitted to; zero-order equation, Q_t_ = Q_o_ + K_o_ t; first-order equation^13^,^14^ Q_t_ = Q_o_ e^−Kt^; and Higuchi's^15^ square root model Q_t_ = K_H_√t, where, Q_t_ is the amount of drug released in time‘t’, Q_o_ is the initial amount of drug in dissolution medium, and K_o_, K and K_H_ are respective release constants. The mechanism of drug release was further analysed using the Korsmeyer-Peppas power law^16^,^17^ M_t_ /M_∞_ = Kt^n^, where, M_t_ /M_∞_ is the fraction of drug released in time ‘t’, K is structural and geometric constant, and n is the release exponent.

### Statistical analysis:

All data were analysed by Student's t-test and one-way ANOVA, wherever necessary, using Sigma Stat 2.0 (Jandel Scientific corporation, USA) to determine the statistical difference in the results. A probability value *p* < 0.05 was considered statistically significant.

## RESULTS AND DISCUSSION

Chitosan, being a multivalent cationic polysaccharide forms gel with suitable anions. Sodium tripolyphosphate was used in this study as a counter ion for chitosan. Chitosan is insoluble in alkaline and neutral pH, but soluble in acidic solvents. The amine groups undergo protonation in acidic environment that increases its solubility in acidic solutions^18^. The protonated amine groups interact with phosphate ions, provided by TPP, either by intermolecular or intramolecular linkage. On addition of chitosan solution to TPP solution, the acetic acid of chitosan solution is rapidly neutralized with the ingress of coagulation fluid which causes decrease in pH within the beads^7^. Simultaneously, the amine group of chitosan interacts with phosphate ions of tripolyphosphate solution. Both processes lead to precipitation of chitosan with simultaneous entrapment of added ciprofloxacin within its matrix.

**Fig. 1:**
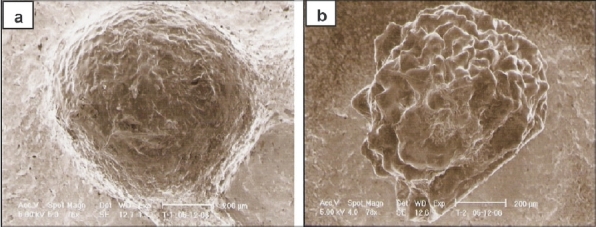
Surface SEM images of the prepared beads. (a) placebo chitosan beads and (b) Ciprofloxacin-loaded beads (batch CB2).

The size of both wet and dry beads were determined and recorded (Table 1). Wet beads were in the range of 2.08±0.51 – 2.31±0.24 mm. On drying, the size of beads decreased significantly (*p* < 0.05) to 0.60±0.09 – 0.70±0.03 mm. The large size of wet beads suggests of high swelling and water retention ability of the beads. Bead size increased markedly with an increase in the drug loading. SEM imaging revealed that beads (without ciprofloxacin) were spherical, with rough and wrinkled surface (fig. 1a). However, the shape of the ciprofloxacin loaded beads was nearly spherical but with non-uniform surfaces (fig. 1b).

Encapsulation efficiency of the multi-unit systems is an important parameter. As the curing occurs in aqueous solution, it is likely that a part of the drug would leach into coagulation fluid. The curing duration had an impact on the entrapment efficiency of fabricated beads. ciprofloxacin entrapment decreases as the curing time increases. Highest drug entrapment, 90±1.96%, was achieved at a short curing time (10 min). However, this time was insufficient for complete gelation of chitosan^7^. The minimum curing time determined for complete gelation of chitosan beads with satisfactory encapsulation was 20 min. In coagulation fluid, acetic acid diffuses into the external phase and the TPP solution diffuse into the droplets. During this process, the added drug diffuses to the external phase, hence, longer retention of the beads in the coagulation fluid lowers drug entrapment. The order of encapsulation was 90±1.96, 69.46±2.86, 61.85±2.45, 40.02±2.01 and 27.93±1.94% in 10, 20, 30, 45 and 60 min, respectively.

Water uptake capacity of the beads was determined in two different media and varied with pH of the media used. Higher water uptake was observed in simulated gastric fluid than in simulated intestinal fluid. The phenomenon is expected, based on the pH dependent solubility of chitosan. Other than pH of the media, the water uptake of bead was influenced by pH of TPP solution. The swelling ratio was higher for chitosan beads prepared in TPP solution at pH 5.0 than when the pH was 8.5. Amongst the two media used, swelling ratio was high at pH 1.2 in comparison to 7.4 which is in line with the earlier report by Mi *et al.*^19^.

The degradation rate of the beads depended on the pH of test medium. In acidic medium (0.1 N HCl, pH 1.2) the degradation was faster with >50% mass loss in ∼5.5 h. Conversely, the degradation was found to be negligible at pH 7.4. The faster degradation in SGF was in contrast with the observations of Durkut *et al.*^20^, wherein the complete degradation was achieved in 6 months. The observed faster degradation could be due to high acid solubility of ciprofloxacin, which caused pores in the matrix leading to easy ingress of SGF and subsequent degradation.

**Fig. 2:**
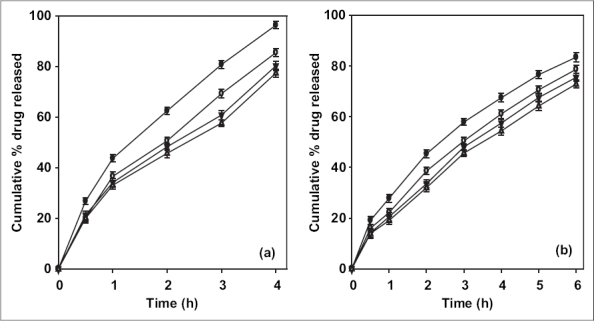
Effect of pH of dissolution medium on *in vitro* ciprofloxacin release. Drug release profiles of CB1 (–●–), CB2 (–○–), CB3 (–▼–) and CB4 (–△–) in (a) SGF (0.1 N HCl, pH 1.2) (b) pH 7.4.

**Fig. 3:**
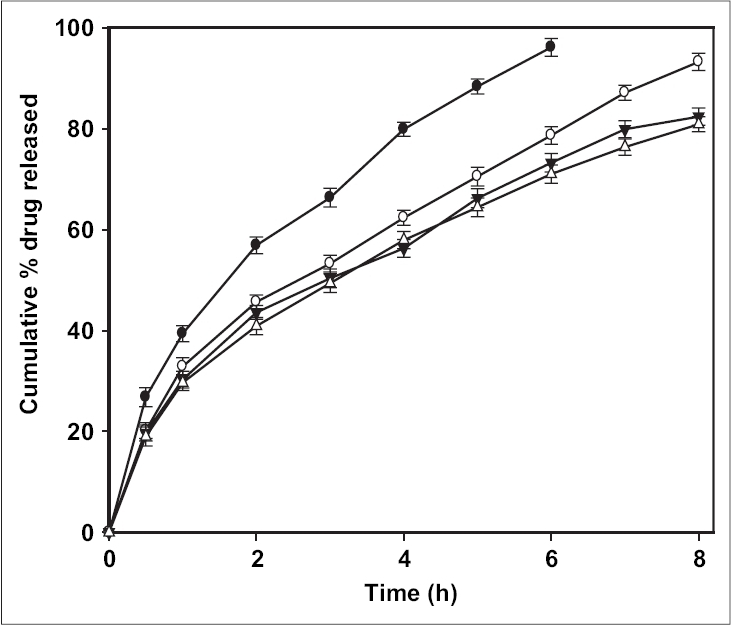
Effect of change of pH of dissolution fluid on drug release from the beads. *In vitro* drug release was tested for first two hours in pH 1.2 (0.1 N HCl) and changed to pH 7.4. CB1 (–●–), CB2 (–○–), CB3 (–▼–), CB4 (–△–).

The release of ciprofloxacin in two different media (pH 1.2 and 7.4) is shown in fig. 2. The percent of drug released at pH 1.2 was higher in comparison to that at pH 7.4 exhibiting pH-sensitivity of the beads. At the end of 4 h, 98% of drug load was depleted from CB1 (TPP- 1% w/v) in SGF (0.1 N HCl, pH 1.2), in comparison to 70% in SIF (pH 7.4). The result was similar for beads prepared with higher concentration of TPP with a release of ∼70% for CB4 (TPP- 4% w/v) at pH 1.2 and ∼50% at pH 7.4. The results indicate significant (*p* < 0.05) influence of pH of dissolution medium on drug release. The faster drug release at pH 1.2 and sustained release at pH 7.4 corroborates with the results of water uptake and degradation studies. An initial burst release of drug was observed from all the batches that can be attributed to two reasons: the leaching of drug on the bead outer surface and faster ingress of dissolution medium and subsequent diffusion of drug. However, on changing the pH from lower to higher level, the drug release slowed (fig. 3). At the end of 8 h, 93.2% of drug was released from CB2 in comparison to 85.4% in 4 h at pH 1.2. Similar pattern was observed in the case of CB3 and CB4. The exception being CB1, which even with pH change released 96% drug in 6 h (the corresponding release in pH 1.2 was 96.3% in 4 h). The results are in contrast to the earlier report of poor pH-responsive drug release of riboflavin^21^, but similar to other researchers^22^,^23^. The pH-responsive release can best be explained based on charge density on the beads^19^, which is an important factor in electrostatic interaction and depends on solution pH. In SGF, protonation of phosphate ions causes hydrogen bond to break, leading to weaker electrostatic interaction. This caused swelling and higher release in SGF, while in SIF stronger attractive force between phosphate ions and chitosan caused slower release of drug. The change in pH of dissolution medium caused swelling (2 h, pH 1.2) and later deswelling (in pH 7.4) leading to bimodal drug release.

Sodium tripolyphosphate was used at four concentrations to investigate its effect on the release profile. Drug release from hydrogels is controlled by the degree of cross-linking^24^. The release of ciprofloxacin from the beads decreased with increased cross-linking agent concentration. However, except for 1% w/v TPP, the drug release did not vary significantly (*p* > 0.05). The primary reason for this observation is, increasing the cross-linking density reduces swelling of the beads hindering drug release^7^.

**Fig. 4:**
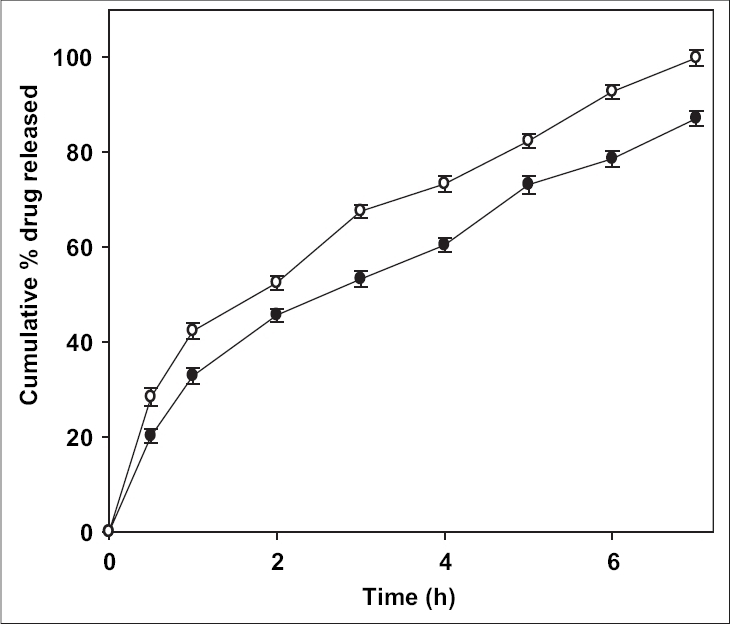
Effect of pH of coagulation fluid on drug release. Beads were fabricated with coagulation fluid (sodium tripolyphosphate solution) at pH 5 (–●–) and 8.5(–○–).

The effect of pH of TPP solution on drug release is shown in fig. 4. Ciprofloxacin release from the beads was influenced by the pH of TPP solution. Beads prepared in original solution (pH 8.5) released ciprofloxacin faster than those prepared after adjusting the pH of TPP solution to 5.0. The pore structure of chitosan microparticle was modified by the change in the pH of TPP solution^25^. At higher pH open porous structure with low density was reported by Ko *et al.*^26^. The ionization of amine group on chitosan decreases with increasing pH proceeding to weaker cross-liking density. These reasons attribute to the faster release of ciprofloxacin from beads prepared in TPP solution at pH 8.5.

**Fig. 5:**
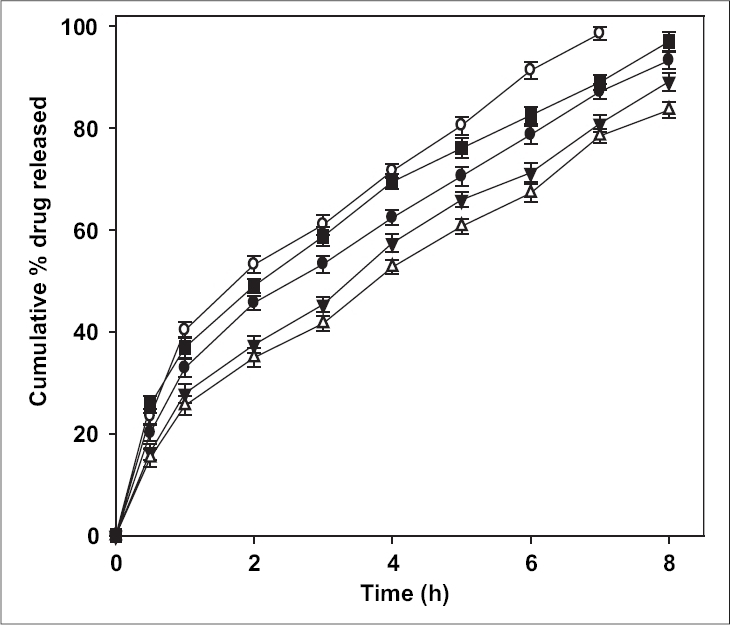
Influence of ciprofloxacin and chitosan concentration on *in vitro* drug release profile. Drug release profile of batches CB2 (–●–), CB5 (–○–), CB6 (–▼–), CB7 (–△–) and CB8(–■–).

The release of ciprofloxacin depends on its concentration in the bead and chitosan (fig. 5). Fast and complete drug release was observed from CB5 and CB8 containing equal ratio of ciprofloxacin and chitosan (1:1 drug:chitosan) in comparison to other batches. The drug release was reduced with decrease in ciprofloxacin and increase in chitosan concentration. Beads exhibited a burst release (∼20% in 30 min). The diffusion of drug from the surface creates a pore in the matrix which causes a channelling effect. Incorporation of higher concentration of drug causes more pore formation leading to faster and higher drug release. This phenomenon was in contrast to the reported decrease in drug release with increasing concentration of sulphadiazine, a poorly soluble drug^7^.

The regression co-efficient (r^2^) for equation Q_t_ = Q_o_ e^−Kt^ was between 0.9902 and 0.9968 for batches CB1, CB2, CB3 and CB7. This was higher than for other kinetic equations indicating that drug release followed first-order kinetics. Other fabricated batches viz., CB4, CB5, CB6 and CB8, showed higher regression co-efficient (between 0.9853 and 0.9979) for Higuchi's square root equation (Q_t_ = K_H_√t) indicating time dependent release mechanism. The release exponent (n) values (Table 1) were in the range of 0.47-0.59 suggesting drug release by a combination of diffusion and dissolution. The observed deviation from Fickian mechanism could be possibly due to higher molecular weight^27^ of ciprofloxacin (331.4) and the polymer characteristics (solubility, pKa, tortuosity)^28^. The fabricated beads showed good reproducibility. The process being simple can be used in the formulation of sustained release formulations. A desired release profile could be achieved by modifying a few process parameters, which are discussed above. Further studies are needed to evaluate the performance of these systems in *in vivo* and to optimize the formulation.
